# Impact of the 2019 Coronavirus Disease Pandemic on Health-Related Quality of Life and Psychological Status: The Role of Physical Activity

**DOI:** 10.3390/ijerph18083992

**Published:** 2021-04-10

**Authors:** Hosam Alzahrani, Fahad Alshehri, Muhsen Alsufiany, Hatem H. Allam, Rania Almeheyawi, Marwa M. Eid, Kabir P. Sadarangani

**Affiliations:** 1 Department of Physical Therapy, College of Applied Medical Sciences, Taif University, Taif 21944, Saudi Arabia; ifahad81@gmail.com (F.A.); muhsen_pt@hotmail.com (M.A.); hatem.lamiaa@gmail.com (H.H.A.); ralmeheyawi@tu.edu.sa (R.A.); alaaamr50@yahoo.com (M.M.E.); 2 Department of Physical Therapy for Surgery, Faculty of Physical Therapy, Cairo University, Cairo 12613, Egypt; 3 Escuela de Kinesiología, Facultad de Salud y Odontología, Universidad Diego Portales, Santiago 8370057, Chile; kabir.sadarangani@gmail.com; 4 Universidad Autónoma de Chile, Santiago 7500912, Chile

**Keywords:** COVID-19, coronavirus, pandemic, impact, psychological stress, active, health

## Abstract

This study investigated the impact of the 2019 coronavirus disease (COVID-19) pandemic on health-related quality of life (HRQoL) and psychological status among Saudi adults, and whether physical activity modifies this association. The participants were 518 adults aged ≥18 years (67.4% men). Using an online survey, data regarding demographic information, the impact of COVID-19 (assessed by the Posttraumatic Stress Disorder Checklist for Diagnostic and Statistical Manual of Mental Disorders-5), HRQoL (Short Form-8), psychological distress (Depression, Anxiety and Stress Scale), and physical activity behavior (International Physical Activity Questionnaire-Short Form) were collected. The results demonstrate that adults reporting moderate or high levels of impact of COVID-19 had a lower HRQoL and higher psychological distress than adults reporting a low impact. HRQoL was higher for adults reporting any level impact (low, moderate, or high) of COVID-19 when they participated in recommended levels of physical activity (≥600 metabolic equivalent (MET)-min/week of total physical activity). Psychological distress was lower for adults reporting a high level of impact when they participated in recommended physical activity. Moderate or high levels of impact of COVID-19 were associated with a significantly lower HRQoL and higher psychological distress than the low impact of COVID-19. However, these associations were moderated by the recommended levels of physical activity.

## 1. Introduction

The 2019 coronavirus disease (COVID-19) pandemic is a serious threat to public health, reported as the biggest outbreak of atypical pneumonia since the severe acute respiratory syndrome (SARS) outbreak in 2003 [1]. The overall number of cases and deaths within a few weeks at the beginning of the outbreak surpassed SARS [2,3]. As such, COVID-19 has become a pandemic, spreading rapidly outside China. On 30 January 2020, the World Health Organization (WHO) declared the COVID-19 outbreak a public health emergency of international concern [4].

In response to the COVID-19 pandemic, countries worldwide have adopted numerous safety measures to prevent its spread [5]. In Saudi Arabia, these strategies include the closure of educational institutions and malls, restrictions on travel and sporting activities, prevention of social gatherings, and imposing a partial or complete lockdown [6]. The partial (usually from 3 pm to 6 am) or complete (24 h) lockdown lasted for around three months starting from mid-March to the end of June [7]. As a result, people may spend most of their time at home watching television or playing video games, which may have a detrimental impact on their physical activity behavior, consequently resulting in deterioration of the overall health status [8,9]. 

Health-related quality of life (HRQoL) is a multidimensional concept which is widely used as a positive health indicator and could be used as a useful indicator of the impact and consequences of the COVID-19 pandemic on peoples’ satisfaction with physical, social and psychological functioning [10]. Previous studies conducted in Saudi Arabia have shown that COVID-19 has had a negative impact on various dimensions of the QoL and psychological health of the population [11,12]. The detrimental impact of psychological health disorders on HRQoL has been well documented in population-based studies [13,14]. Moreover, lower HRQoL is associated with greater morbidity and mortality [15,16]. 

Various psychological responses to the outbreak may arise, including fear and panic of being infected, concerns regarding personal health and the health of their family, relatives, and friends, stress, anxiety, and depression [17,18]. A recently published study conducted during the initial stage of the COVID-19 outbreak showed that the majority of participants reported a moderate to severe psychological impact [19,20]. This could be explained by the spread of the epidemic, associated severe symptoms, and the consequent higher mortality rate. However, there is still limited data on the impact of COVID-19 on the psychological health status of the general population. Furthermore, most of the published research related to COVID-19 focused on infected patients or those with suspected COVID-19 symptoms. 

Together, these results suggest the need for the implementation of intervention strategies, especially for people who are at higher risk. One such evidence-based strategy is to increase physical activity in the community. Physical activity is a key requirement for improving health and HRQoL [21,22]. Currently, the WHO recommends that adults perform at least 600 (or 3000 for extra benefit) metabolic equivalent minutes (METs) of total physical activity throughout the week (MET-min/week) [23]. 

The overall aims of this study were to (1) determine whether the impact of the COVID-19 pandemic is independently related to HRQoL and psychological status among adults in Saudi Arabia, and (2) determine whether these associations vary across different levels of physical activity.

## 2. Materials and Methods

### 2.1. Study Design

This cross-sectional study was conducted in June 2020. Only adult participants (aged 18 years and older) living in Saudi Arabia during the COVID-19 pandemic period were included in the study. Participants completed an anonymous, confidential online questionnaire in Arabic through an online survey platform (Google Forms). Participants received an announcement message including a summary of the study and question regarding whether they agree to participate in the study. If they agree to participate in the study, they are directly forwarded to the next page which includes the survey questions. The questionnaire was advertised and distributed by researchers through social networks (e.g., Twitter, Facebook and WhatsApp) as it was the most feasible way to reach target participants in light of social-distancing protocols being implemented, and lockdown enforced, during the COVID-19 pandemic. Furthermore, this method of circulating questionnaires through social networks was used to easily access and maximize the reach of diverse participants from different regions in Saudi Arabia. Participants were also asked to circulate the survey to their relatives and professional networks. A non-random snowball sampling method was used to select participants. Participation in the study was voluntary and participants were not offered any incentives. The study protocol was approved by the institutional review board of Taif University (Application No. 41-00184). This study was reported according to Strengthening the Reporting of Observational Studies in Epidemiology (STROBE) recommendations (Supplementary File S1) [24].

### 2.2. Descriptive Statistics

According to a statistical report conducted by the General Authority for Statistics in Saudi Arabia (GASTAT) in 2018, the total number of adult populations was approximately 25,000,000. The sample size was calculated by setting the statistical power at a 95% confidence interval (CI), with a population size of 25,000,000 and a margin of error of 5%. Thus, the sample size required for this study was 385 participants.

A total of 518 questionnaires were completed and received. The general characteristics of the included participants, grouped by physical activity level, are presented in Table 1. The average age was 37.3 ± 14.3 years. The 518 participants included 349 (67.4%) males and 169 (32.6%) females. Approximately 97.7% were Saudi, 31.9% had normal weight, 31.9% were overweight, 94.6% had a high school or below education, 61.6% were married, 57.1% were unemployed, 36.7% had a monthly income of 10,000–20,000, 72.0% had never smoked, and 66.6% had no chronic diseases. According to geographical division, most participants were from the central (38.6%) and western (37.8%) regions. There were nine participants (1.7%) who were infected with COVID-19 and 194 participants (37.5%) who had an infected relative. Sufficiently active participants comprised 43.8% of the sample, their average age was 38.2 ± 14.7 years, and they were more likely males, overweight, and had never smoked.

### 2.3. Survey Development and Instruments

The structural questionnaire consisted of questions related to the following areas: (1) socio-demographic information, (2) impact of COVID-19, (3) psychological status, (4) HRQoL, and (5) physical activity behavior. The questionnaire also included questions related to COVID-19. 

Sociodemographic data included age, sex, height, weight, ethnicity, smoking, education, region, marital status, employment status, social status, occupation, and income. Information on current health status and disease history, including chronic diseases, was also collected. 

The impact of COVID-19 was assessed using the Posttraumatic Stress Disorder Checklist for Diagnostic and Statistical Manual of Mental Disorders (DSM)-5 (PCL-5) [25,26]. The PCL-5 is a 20-item self-report questionnaire that evaluates the presence and severity of posttraumatic stress symptoms, or the impact of life stress or unexpected events in the past month. The PCL-5 has been well validated in the Arab population to assess the impact of a traumatic or distressing event [27]. Responses for each item are rated on a 5-point Likert scale (0 = “Not at all” to 4 = “Extremely”). An overall symptom severity score (range: 0–80) is obtained by summing the scores of all items, with a higher score indicating a higher level or impact of posttraumatic stress. The PCL-5 variable was categorized into levels framed around tertiles: (a) low impact (reporting < 11), (b) moderate impact (≥11 to <22), and (c) high impact (reporting ≥ 22). 

HRQoL refers to how health impacts people’s ability to function and their perceived well-being in physical, psychological, and social domains of life. HRQoL was measured using the Short Form-8 Item (SF-8) [28]. The SF-8 is an eight-item scale that assesses physical functioning, role physical, bodily pain, general health, vitality, social functioning, role emotional, and mental health. A total percentage score was calculated for each of these domains, ranging from 0 (lowest or worst status) to 100 (highest or best status). We calculated the overall score of HRQoL by taking the average of the eight domains (range: 0–100), with higher scores indicating better health. The SF-8 was developed to replicate the SF-36, and it has been shown to have a high test-retest reliability and discriminant validity for assessing HRQoL in the general population [29,30,31,32].

Psychological status was measured using the Depression, Anxiety and Stress Scale-9 (DASS-9) [33,34]. The DASS-9 is a modified and a shortened version of the DASS-21 instrument and is composed of nine questions evaluating three subdomains (depression, anxiety, and stress) and has the same structure as the original full version [35,36]. Responses for each question are scored on an answer scale of four points (0 = “not at all” to 3 = “most of the time”), with higher scores indicating a greater level of psychological distress (range: 0–27). The DASS-9 has been shown to have sound psychometric properties comparable to those of DASS-21 [33,34]. Therefore, we used the same translation for the nine questions in the Arabic DASS-21 version [37]. The scores were calculated based on previous studies [34,37,38,39]. The DASS-9 has been shown to have adequate reliability and convergent validity for assessing psychological health in the population [33,38,39].

Physical activity level was assessed using the Arabic version of the International Physical Activity Questionnaire-Short Form (IPAQ-SF) which is available in the IPAQ official website (www.ipaq.ki.se (accessed on 5 April 2020)) [40]. The IPAQ has been validated in 12 countries and translated into several languages, including Arabic [40,41]. Furthermore, the Arabic Version of IPAQ demonstrated an acceptable validity and reliability for the assessment of physical activity in the Arab population [41]. Concurrent validity (inter-method) coefficients between IPAQ short and long forms have reasonable agreement (*p* = 0.67; 95% CI 0.64, 0.70) [39]. The IPAQ-SF asks participants about their time spent (days per week, and minutes per day) over the last seven days on three different intensities of physical activity (vigorous (8 MET), moderate (4 MET), walking (3.3 MET)) for at least 10 min at a time [40]. The overall physical activity score was calculated using an MET task scored in minutes per week (MET-min/week) [40,42]. The physical activity data (expressed in MET-min/week) were reported as a categorical score: inactive (<600), sufficiently active (≥600), and very active (≥3000), and as a continuous score (https://sites.google.com/site/theipaq/ (accessed on 5 April 2020)). 

### 2.4. Statistical Analysis

Statistical analyses were conducted using SPSS Statistics software (version 23.0; IBM Corp., Armonk, NY, USA). Descriptive data were reported as either mean ± standard deviation (SD) or frequency and percentage (%). Continuous variables, SF-8 and DASS-9, were tested for outliers, and there were no extreme values identified with their distribution. 

The first analysis was to investigate the extent to which the COVID-19 impact (PCL-5) was associated with HRQoL (SF-8) and psychological distress (DASS-9). It was conducted using generalized linear models and multiple linear regression to determine linear trend *p*-values. Generalized linear model coefficients indicate mean differences in HRQoL and psychological distress between the reference category (low) and each of the other COVID-19 impact variables. We also stratified the analysis of COVID-19 impact on HRQoL and psychological distress by sex (male and female). An effect size (Cohen’s d) for this analysis was calculated following previous published methods [43]. 

The second analysis was conducted to investigate the associations between physical activity and HRQoL or psychological distress within each level of COVID-19 impact category. The analysis was conducted using generalized linear models and multiple linear regression to determine linear trend *p*-values.

The analyses were adjusted for age, sex, body mass index (BMI), ethnicity, the region of residence, social status, education, employment, income, smoking status, and chronic diseases. We reported unstandardized beta coefficients in all regression models. All statistical significance tests were set at a *p*-value of <0.05. 

## 3. Results

The results of the multivariable-adjusted linear regression analysis for the impact of COVID-19 on HRQoL are shown in Table 2. The impact levels were independently associated with HRQoL (*p* < 0.001). The results demonstrate that adults reporting moderate or high levels of impact had a significantly lower HRQoL (coefficient −10.19, 95% CI −13.18, −7.21, and coefficient −20.39, 95% CI −23.47, −17.32, respectively) than adults reporting a low level of impact. 

In the stratified analysis by sex, the results show that the HRQoL associated with COVID-19 impact had a similar trend between males and females (Supplementary Table S1).

The results of the multivariable-adjusted linear regression analysis for the impact of COVID-19 on psychological distress are shown in Table 2. The level of impact was independently associated with psychological distress (*p* < 0.001). The results demonstrate that adults reporting moderate or high levels of impact had a significantly higher level of psychological distress (coefficient 11.89, 95% CI 7.46, 16.32, and coefficient 39.77, 95% CI 35.21, 44.33, respectively) than adults reporting a low level of impact. 

In the stratified analysis by sex, the results demonstrate that the psychological distress associated with COVID-19 impact was more pronounced in females than in males (Supplementary Table S1).

**Figure 1:**
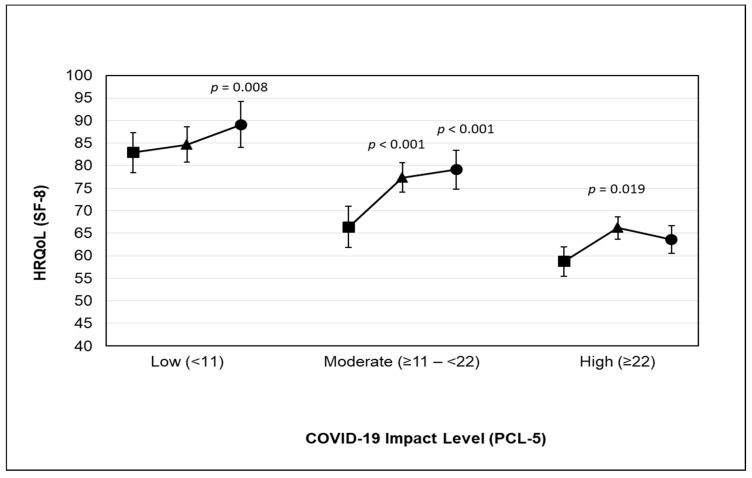
Predicted marginal mean HRQoL for COVID-19 impact among adults participating in physical activity ((■ Inactive: reporting <600 MET-min/week), (▲ sufficiently active: reporting ≥600 MET-min/week), and (● very active: reporting ≥3000 MET-min/week)). The model was adjusted for age, sex, body mass index, ethnicity, region of residence, social status, education, employment, income, smoking status, and chronic diseases. Abbreviations: CI, confidence interval; HRQoL, health-related quality of life; PCL-5, posttraumatic stress disorder checklist; SF-8, short form-8 item.

The predicted marginal mean HRQoL for COVID-19 impact among adults participating in various physical activity levels is shown in Figure 1. HRQoL for adults reporting a low level of impact was higher when they participated in at least 3000 MET-min/week of total physical activity (predicted marginal mean HRQoL = 89.13) than when they engaged in less than 600 MET-min/week of total physical activity. 

HRQoL for adults reporting a moderate level of impact was higher when they participated in at least 600 and 3000 MET-min/week of total physical activity (predicted marginal mean HRQoL = 77.34, and HRQoL = 79.08, respectively) than when they engaged in less than 600 MET-min/week of total physical activity. 

HRQoL for adults reporting a high level of impact was higher when they participated in at least 600 MET-min/week of total physical activity (predicted marginal mean HRQoL = 66.15) than when they engaged in less than 600 MET-min/week of total physical activity. 

**Figure 2:**
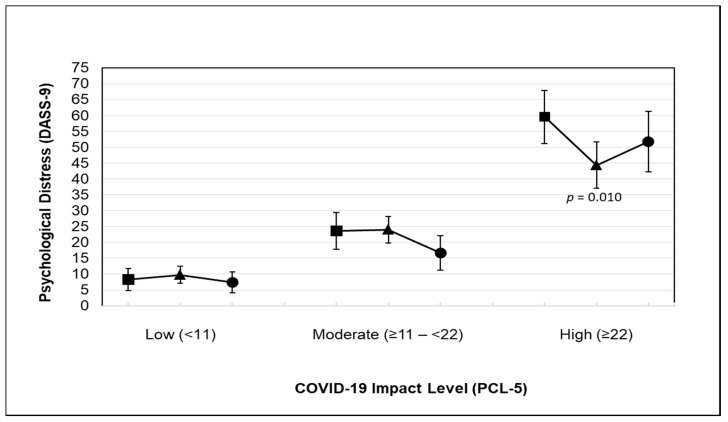
Predicted marginal mean psychological distress for COVID-19 impact among adults participating in physical activity ((■ Inactive: reporting <600 MET-min/week), (▲ sufficiently active: reporting ≥600 MET-min/week), and (● very active: reporting ≥3000 MET-min/week)). The model was adjusted for age, sex, body mass index, ethnicity, region of residence, social status, education, employment, income, smoking status, and chronic diseases. Abbreviations: CI, confidence interval; DASS-9, Depression, Anxiety, and Stress Scale; PCL-5, posttraumatic stress disorder checklist.

The predicted marginal mean psychological distress for COVID-19 impact among adults participating in various physical activity levels is shown in Figure 2. Psychological distress for adults reporting a high level of impact was lower when they participated in at least 600 MET-min/week of total physical activity (predicted marginal mean psychological distress = 44.31) than when they engaged in less than 600 MET-min/week of total physical activity. 

## 4. Discussion

The results of this study demonstrate that adults reporting moderate or high impact levels of COVID-19 showed significantly lower HRQoL and higher psychological distress. However, these associations were moderated by the level of physical activity.

Concerning the association between COVID-19 impact and HRQoL, the results of our study reveal that the higher the COVID-19 impact level, the lower the HRQoL. Adults reporting moderate or high impact levels had a significantly lower HRQoL than adults reporting a low impact level. These findings are in agreement with Nguyen et al. [44], who found that people with suspected COVID-19 had lower HRQoL. Furthermore, a previous study conducted in Saudi Arabia during the COVID-19 pandemic found that COVID-19 had a significant impact on various aspects of QoL [11]. These findings may be attributed to some preventive measures and protocols that were followed because of the COVID-19 pandemic, such as travel constraints, staying at home for longer periods, decreasing leisure activities, and self-isolation, which worsens the QoL and decreases the level of life satisfaction.

Concerning the impact of COVID-19 on psychological status, the results reveal that adults reporting moderate or high impact levels had significantly higher psychological distress than adults reporting low impact levels. Our results agree with Solomou and Constantinidou [45], who found that COVID-19 had a great impact on the psychological status and quality of life (QOL) of the general population. They explained that the increased psychological distress might be due to the coincidence of specific factors during the pandemic, such as fear of high mortality rate and even health consequences after recovery, financial instability, insecurity regarding job stability, insufficient social support, length of isolation time, and high exposure to the media (particularly problematic social media). Additionally, Satici et al. [46] agreed with the conclusion that there was a significant positive correlation between fear of COVID-19 and psychological distress. They related a higher degree of psychological distress to several factors, including continuous exposure to news reporting wide-reaching mortalities or the infection rate of COVID-19.

Stratified analysis according to sex demonstrated that the association between COVID-19 impact and HRQoL had a similar trend among males and females. However, the association between COVID-19 impact and psychological distress was more evident among females than in males. This finding was in line with previous studies conducted in China and the UK during the COVID-19 pandemic which showed that the females’ psychological distress was greater than that of males [47,48]. These studies attributed that to some potential reasons, including increasing responsibilities at home and the long hours spent on childcare during the pandemic, in addition to other sex differences such as psychodynamic, cognitive and social environmental processes. Further studies, however, are still needed to understand the mechanisms underlying these observed differences in psychological distress between males and females. 

Regarding the role of physical activity in moderating the association between COVID-19 impact and HRQoL, the results of the study reveal that, regardless of the impact level, there was a significant increase in HRQoL in highly active and sufficiently active participants compared with inactive participants. These findings agree with the findings of previously published studies [49,50] which suggested that people who reported high physical activity participation had a better QoL. Moreover, Nguyen et al. [44] agreed with our findings, as they mentioned that there was a positive association between physical activity and HRQoL. Furthermore, a recent study conducted by Esain et al. [51] showed that three months of getting off of physical activity led to a deterioration in QoL and mental health in physically active populations. Another study published by Nayak et al. [52] found that the HRQoL in Chinese adults worsened during the pandemic. They attributed these findings to a decrease in physical activity and a prolonged sedentary lifestyle. Furthermore, they reported that highly active people had significantly better HRQoL and reduced levels of distress. Increasing physical activity level could help in transitioning people to a healthier lifestyle and consequently improving HRQoL. 

Our results reveal that the association between COVID-19 impact and psychological distress was moderated by the level of physical activity. Psychological distress for adults reporting a high level of impact of COVID-19 was lower in sufficiently active and highly active participants than in inactive participants. Moreover, psychological distress in adults reporting a moderate impact level of COVID-19 was lower in highly active participants than in inactive participants. These findings agree with those of Pieh et al. [53], who concluded that engaging in a high level of physical activity was associated with lower psychological distress in the general population. Additionally, Nguyen et al. [44] confirmed our findings, as they reported that the incidence of depression was significantly lower in participants who were more physically active than those who were less active. Therefore, current physical activity guidelines should be established and encouraged during the pandemic to promote healthy lifestyles.

One mechanism that may explain the positive role of physical activity in improving the poor HRQoL and worse psychological health attained by COVID-19 impact and lockdown is that engaging in outside physical activities and exposure to nature improves psychological status, happiness and mood, and decreases feeling of anger, confusion and tension [54,55]. Furthermore, improving in these mentioned factors can lead to improving in HRQoL dimensions related to these variables (e.g., mental health, role emotional, vitality, social functioning and general health), which will then be reflected in the overall HRQoL. 

To the best of our knowledge, this is the first study to assess the role of physical activity in moderating the associations between the COVID-19 pandemic and HRQoL and psychological status in the general population. One strength of the current study is achieving the required sample size, which was adequate and more powerful in exploring associations and detecting interactions. Another strength is the availability of data on comorbidities (including cardiovascular and respiratory disease, diabetes, neurological disease, gastrointestinal disease, headache, hematologic disease, endocrine disease, cancer, and musculoskeletal disease), which enabled us to adjust for the prevalent chronic diseases, which are considered important potential confounders in the association of COVID-19 impact with HRQoL and psychological distress. 

There were some limitations of the current study. First, although we used a convenience sampling method to easily access and reach large diverse participants from different regions in Saudi Arabia, this sampling method might have some risk of bias and limit the generalizability of results. Second, causal relationships could not be established, as the design of this study was cross-sectional. Therefore, a prospective longitudinal study is required. Third, the use of questionnaires might be subject to recall bias, which may result in participants underestimating or overestimating their response, for example, activity level [56]. Nonetheless, this potential bias may be mitigated to some degree by the acceptable convergent validity of the questionnaires used in this study. 

## 5. Conclusions

In conclusion, the results of this study found that the impact levels of COVID-19 were independently associated with HRQoL and psychological distress. However, these associations were moderated by the recommended levels of physical activity. Therefore, future studies should consider a longitudinal study design to establish a cause-and-effect relationship between the impact of COVID-19 on adults and their HRQoL and psychological status.

## Figures and Tables

**Table 1:** Demographics and health-related characteristics of participants.

Characteristics	All Participants (*n* = 518)	Physical Activity (MET-Min/Week)			
		Inactive (*n* = 149)	Sufficiently Active (*n* = 227)	Very Active (*n* = 142)	*p* Value *
	No. (%)	%	%	%	
**Age (years), mean (SD)**	37.3 (14.3)	37.4 (14.3)	38.2 (14.7)	35.7 (13.6)	0.023
**Sex**					0.161
Male	349 (67.4)	72.5	67.4	62.0	
Female	169 (32.6)	27.5	32.6	38.0	
**Ethnicity**					0.538
Saudi	506 (97.7)	96.6	97.8	98.6	
Non-Saudi	12 (2.3)	3.4	2.2	1.4	
**BMI, kg/m^2^**					0.092
Underweight (<18.5)	36 (6.9)	11.4	6.2	3.5	
Normal (18.5 to 24.9)	165 (31.9)	26.2	32.2	37.3	
Overweight (25 to 30)	165 (31.9)	30.2	33.9	30.3	
Obese (≥30)	152 (29.3)	32.2	27.8	28.9	
**Smoking status**					0.342
Never smoker	373 (72.0)	69.8	75.8	68.3	
Previous smoker	48 (9.3)	8.7	7.5	12.7	
Current smoker	97 (18.7)	21.5	16.7	19.0	
**Highest level of education**					0.410
High school or below	490 (94.6)	94.0	96.0	93.0	
Bachelor or above	28 (5.4)	6.0	4.0	7.0	
**Employment status**					0.170
Currently employed	222 (42.9)	49.0	39.2	42.3	
Not employed	296 (57.1)	51.0	60.8	57.7	
**Social status**					0.048
Single	188 (36.3)	32.9	35.7	40.8	
Married	319 (61.6)	67.1	60.4	57.7	
Widow/divorced	11 (2.1)	0.0	4.0	1.4	
**Monthly income**					<0.001
None	101 (19.5)	14.1	17.2	28.9	
<6000	123 (23.7)	27.5	24.2	19.0	
6000–10,000	55 (10.6)	4.7	14.5	10.6	
10,000–20,000	190 (36.7)	36.2	37.9	35.2	
>20,000	49 (9.5)	17.4	6.2	6.3	
**Region of residence**					0.043
Central region	200 (38.6)	46.3	33.0	39.4	
Western region	196 (37.8)	37.6	38.3	37.3	
Eastern region	44 (8.5)	6.0	11.5	6.3	
Southern region	63 (12.2)	6.7	15.4	12.7	
Northern region	15 (2.9)	3.4	1.8	4.2	
**Prevalent chronic diseases**					<0.001
No	345 (66.6)	55.0	66.1	79.6	
Yes	173 (33.4)	45.0	33.9	20.4	
**Self-infection with COVID-19**					0.207
No	509 (98.3)	97.3	99.6	97.9	
Yes	9 (1.7)	2.7	0.4	2.1	
**Relative-infection with COVID-19**					0.134
**No**	324 (62.5)	55.0	63.0	62.5	
**Yes**	194 (37.5)	45.0	37.0	37.5	

Abbreviations: BMI, body mass index; MET, metabolic equivalent. Inactive: reporting <600 MET-min/week, sufficiently active: reporting ≥600 MET-min/week and <3000, and very active: reporting ≥3000 MET-min/week. * Chi-squared tests for group differences.

**Table 2:** Multivariable adjusted associations between COVID-19 impact (PCL-5), and HRQoL and psychological distress.

	Predicted Marginal Mean	Coefficient (95% CI) ^a^	Cohen’s d
**HRQoL (SF-8) ^b^**			
Low (*n* = 206)	84.80 (1.00)	Referent	
Moderate (*n* = 151)	74.60 (1.14)	−10.19 (−13.18, −7.21)	−0.55
High (*n* = 161)	64.40 (1.14)	−20.39 (−23.47, −17.32)	−1.03
Trend *p* value		<0.001	
**Psychological Distress (DASS-9) ^b^**			
Low (*n* = 206)	9.85 (1.48)	Referent	
Moderate (*n* = 151)	21.74 (1.69)	11.89 (7.46, 16.32)	0.43
High (*n* = 161)	49.63 (1.69)	39.77 (35.21, 44.33)	1.35
Trend *p* value		<0.001	

Abbreviations: CI, confidence interval; DASS-9, Depression, Anxiety and Stress Scale; HRQoL, health-related quality of life; PCL-5, Posttraumatic Stress Disorder Checklist; SF-8, short form-8 item. Scale range for HRQoL (SF-8): 0–100, higher scores indicative of better status or health. Scale range for psychological distress (DASS-9): higher scores indicative of worst psychological status. Low: reporting <11 on PCL-5 scale; Moderate: 11≤ reporting <22; High: reporting ≥22. ^a^ Model was adjusted for age, sex, body mass index, ethnicity, region of residence, social status, education, employment, income, smoking status and chronic diseases. ^b^ Generalized linear model coefficients; coefficients indicate mean differences (in HRQoL and DASS-9) between the reference category (Low) and each of the other PCL-5 impact severity groups, e.g., a value of 3 indicates that a specific category had a mean score that is 3 units higher than the referent group.

## Data Availability

The data presented in this study are available from the corresponding author on reasonable request.

## Supplementary Materials

The following are available online at https://www.mdpi.com/article/10.3390/ijerph18083992/s1, Table S1: Multivariable adjusted associations between COVID-19 impact (PCL-5), and HRQoL and psychological distress, File S1: STROBE Statement—Checklist of items that should be included in reports of cross-sectional studies.
