# Supplementary material for: Impact of the 2019 Coronavirus Disease Pandemic on Health-Related Quality of Life and Psychological Status: The Role of Physical Activity
Source: Int J Environ Res Public Health. 2021 Apr 10;18(8):3992. doi: 10.3390/ijerph18083992 (PMC8069215; doi:10.3390/ijerph18083992)
Supplement: Supplementary file 1 [file ijerph-18-03992-s001.zip › Supplementary Table S1.docx]

Supplementary Table S1. Multivariable adjusted associations between COVID-19 impact (PCL-5), and HRQoL and psychological distress.

1. Male

|  | |  | |  |
| --- | --- | --- | --- | --- |
|  | **Predicted marginal mean** | | **Coefficient (95% CI) ^a^** | **Cohen’s d** |
| HRQoL (SF-8) ^b^ |  | | |  |
| Low (*n*=155) | 85.64 (1.18) | | Referent |  |
| Moderate (*n*=102) | 76.26 (1.44) | | -9.37 (-13.04, -5.71) | -0.50 |
| High (*n*=92) | 63.65 (1.56) | | -21.99 (-25.93, -18.05) | -1.14 |
| Trend *p* value |  | | < 0.001 |  |
|  |  | |  |  |
| Psychological Distress (DASS-9) ^b^ |  | |  |  |
| Low (*n*=155) | 8.43 (1.72) | | Referent |  |
| Moderate (*n*=102) | 19.36 (2.10) | | 10.93 (5.58, 16.28) | 0.40 |
| High (*n*=92) | 47.06 (2.28) | | 38.64 (32.88, 44.39) | 1.38 |
| Trend *p* value |  | | < 0.001 |  |
|  |  | |  |  |
| Abbreviations: CI, confidence interval; DASS-9, Depression, Anxiety and Stress Scale; HRQoL, health-related quality of life; PCL-5, Posttraumatic Stress Disorder Checklist; SF-8, short form-8 item.  Scale range for HRQoL (SF-8): 0-100, higher scores indicative of better status or health.  Scale range for psychological distress (DASS-9): higher scores indicative of worst psychological status.  Low: reporting < 11 on PCL-5 scale; Moderate: ≥ 11 reporting < 22; High: reporting ≥ 22.  ^a^ Model was adjusted for age, sex, body mass index, ethnicity, region of residence, social status, education, employment, income, smoking status and chronic diseases.  ^b^ Generalized linear model coefficients; coefficients indicate mean differences (in HRQoL and DASS-9) between the reference category (Low) and each of the other PCL-5 impact severity groups, e.g., a value of 3 indicates that a specific category had a mean score that is 3 units higher than the referent group. | | | | |

1. Female

|  | |  | |  |
| --- | --- | --- | --- | --- |
|  | **Predicted marginal mean** | | **Coefficient (95% CI) ^a^** | **Cohen’s d** |
| HRQoL (SF-8) ^b^ |  | | |  |
| Low (*n*=50) | 83.40 (1.91) | | Referent |  |
| Moderate (*n*=49) | 71.57 (1.86) | | -11.84 (-17.21, -6.46) | -0.62 |
| High (*n*=69) | 64.29 (1.57) | | -19.11 (-24.13, -14.09) | -0.90 |
| Trend *p* value |  | | < 0.001 |  |
|  |  | |  |  |
| Psychological Distress (DASS-9) ^b^ |  | |  |  |
| Low (*n*=50) | 10.96 (3.00) | | Referent |  |
| Moderate (*n*=49) | 26.89 (2.93) | | 15.93 (7.48, 24.39) | 0.53 |
| High (*n*=69) | 55.36 (2.48) | | 44.40 (36.51, 52.30) | 1.34 |
| Trend *p* value |  | | < 0.001 |  |
|  |  | |  |  |
| Abbreviations: CI, confidence interval; DASS-9, Depression, Anxiety and Stress Scale; HRQoL, health-related quality of life; PCL-5, Posttraumatic Stress Disorder Checklist; SF-8, short form-8 item.  Scale range for HRQoL (SF-8): 0-100, higher scores indicative of better status or health.  Scale range for psychological distress (DASS-9): higher scores indicative of worst psychological status.  Low: reporting < 11 on PCL-5 scale; Moderate: ≥ 11 reporting < 22; High: reporting ≥ 22.  ^a^ Model was adjusted for age, sex, body mass index, ethnicity, region of residence, social status, education, employment, income, smoking status and chronic diseases.  ^b^ Generalized linear model coefficients; coefficients indicate mean differences (in HRQoL and DASS-9) between the reference category (Low) and each of the other PCL-5 impact severity groups, e.g., a value of 3 indicates that a specific category had a mean score that is 3 units higher than the referent group. | | | | |
